# TUSC2P suppresses the tumor function of esophageal squamous cell carcinoma by regulating TUSC2 expression and correlates with disease prognosis

**DOI:** 10.1186/s12885-018-4804-9

**Published:** 2018-09-15

**Authors:** Fengqiong Liu, Ruijie Gong, Baochang He, Fa Chen, Zhijian Hu

**Affiliations:** 1 Fujian Provincial Key Laboratory of Environment factors and Cancer, School of Public Health, Fujian Medical University, 1 Xuefubei Road, Fuzhou, Fujian 350108 People’s Republic of China; 2 Key Laboratory of Ministry of Education for Gastrointestinal Cancer, Fujian Medical University, 1 Xuefubei Road, Fuzhou, Fujian China; 3 Department of Epidemiology and Health Statistic, School of Public Health, Fujian Medical University, 1 Xuefubei Road, Fuzhou, Fujian China

**Keywords:** Pseudogene, Esophageal squamous cell carcinoma, TUSC2

## Abstract

**Background:**

Pseudogenes are RNA transcripts with high homology with its parent protein-coding genes. Although pseudogenes lost the ability to produce protein, it still exert import biological function, and play important role in the pathogenesis of a wide varity of tumors; However, the role of pseudogenes in esophageal squamous cell carcinoma (ESCC) is poorly understood.

**Methods:**

TUSC2P function in ESCC were explored using both in vitro and in vivo experiments cell proliferation, invasion and apoptosis assay was performed to evaluated the effect of TUSC2P on the tumor biology of ESCC. Expression of relative genes was assessed by quantitative real-time PCR (qRT-PCR) and western blotting in EC109 and TE-1 cell, as well as ESCC patients. 3’UTR luciferase assay was used to confirm the direct binding of miRNAs with TUSC2 and TUSC2P 3’UTR. Relation betweenTUSC2P, TUSC2 and ESCC prognosis was predicted by survival analysis (*n* = 56).

**Results:**

Pseudogene TUSC2P was down regulated in ESCC tissues compared with paired normal adjacent tissues, and the expression of TUSC2P was significantly correlated with survivalof ESCC patients. Over expression of TUSC2P in EC109 and TE-1 cells resulted in altered expression of TUSC2, thus inhibited proliferation, invasion and promoted apoptosis. Dual luciferase assay demonstrated that TUSC2P 3’UTR decoyed miR-17-5p, miR-520a-3p, miR-608, miR-661 from binding to TUSC2.

**Conclusions:**

TUSC2P can suppresses the tumor function of esophageal squamous cell carcinoma by regulating TUSC2 expression and may also serve as a prognostic factor for ESCC patients.

**Electronic supplementary material:**

The online version of this article (10.1186/s12885-018-4804-9) contains supplementary material, which is available to authorized users.

## Background

Esophageal cancer is one of the most leading causes of cancer-related death worldwide, and esophageal squamous cell carcinoma (ESCC) is histologically the most frequent type of esophageal cancer [1]. Esophageal cancer is aggressive and has poor prognosis, and the overall 5-year survival rate of esophageal cancer is about 20% [2, 3]. Additionally, the incidence of ESCC is increasing rapidly [3]. ESCC is a multifactorial, multistage and complex pathological process, which involves the interaction of numerous oncogenes and tumor suppressor genes, as well as transcriptional and post-transcriptional levels of regulation. In the last two decades, non-coding RNA and the importance of its post transcriptional regulation has drawn extensive attentions. The non-coding RNAs family broadened as more new classes of non-coding RNAs have been identified and named, for example lncRNA, miRNA, circRNA, tiRNA etc. [4–7].

For the last few years, depending on the development of large scale sequencing technique and bioinformatics analysis, thousands of pseudogenes were identified [8]. Pseudogenes is a special form of RNA, which resembles mRNA but lost its protein coding function, thus belongs to the family of long non-coding RNA. In most of the cases, pseudogenes are duplicated DNA sequences of its corresponding parent coding gene, and can be transcribed along with other genes into RNA fragments. However the transcripts usually are incomplete or with mutations such as point or frameshift, which resulting in mutant coding-function [9–11]. Interestingly, the RNA fragments derived from pseudogenes can exert biological functions. They play important role in the post-transcriptional regulation at multiple levels including DNA, RNA and protein, and been proven involved in diverse physiological and pathological processes such as carcinogenesis [11, 12]. Though thousands of pseudogenes have been sequenced, very few of them have been functionally characterized so far.

Tumor suppressor candidate-2 (TUSC2) is a novel tumor suppressor gene that found played important role in the pathogenesis of cancer. TUSC2 was firstly reported in a study analyzing frequent deletions in the short arm of chromosome 3p. Frequent deletions in the short arm of chromosome 3p occurs in a wide variety of cancers [13, 14]. Exogenous expression of TUSC2 in non-small cell lung carcinoma cells significantly inhibited tumor cell growth by activating the apoptotic protease activating factor 1 (Apaf-1) [15]. Intravenous systemic delivery of TUSC2 to distant tumors, via intravenous cholesterol nanovesicles, suppressed tumor growth and progression in orthotopic human lung cancer xenograft models [16]. Numerous TUSC2-targeting miRNAs have been predicted by bioinformatics analysis and many targeting has already been validated, which indicating that TUSC2 abundance in cancer cells is largely dependent on post-transcriptional regulation.

Pseudogene of TUSC2 named TUSC2P, sequence of which shared 89% homology with the 3’-UTR of TUSC2. In addition, they shared binding sites of many miRNAs, including miRNA-17-5p, miRNA-608, miRNA-661, miRNA-520a-3p. In this study, we investigated the relationship between TUSC2 and its pseudogene TUSC2P and the potential role of common binding miRNAs in ESCC.

## Methods

### Tissue samples

Fifty-six human esophagus tumor samples were consecutively collected from Zhang Zhou Hospital, and The First Affiliated Hospital of Fujian Medical University between September 2012 and March 2013, and The Fujian Provincial Cancer Hospital between September 2014 and January 2015. Forty-nine oral cancer samples were consecutively collected from The First Affiliated Hospital of Fujian Medical University, within a period from January 2010 to December 2016.

### Construct generation

The TUSC2 and TUSC2P 3’-UTR fragments were synthesized. The synthesized fragments and pcDNA3.1 vector were digested with NheI and ApaI. The digested fragments were then inserted into pcDNA3.1 vector to obtain 3’-UTR over-expression construct.

The fragment of 3’UTR of TUSC2 and TUSC2P which contained potential binding sites of miRNA-17-5p, miRNA-608, miRNA-661, miR-520a-3p were synthesized. The synthesized fragments and psiCHECKTM-2 luciferase vector were digested with XhoI and NotI. The digested fragments were then inserted into the opened psiCHECKTM-2 luciferase vector to obtain the luciferase constructs TUSC2–1, TUSC2–2, TUSC2–3 and TUSC2P-1, TUSC2P-2, TUSC2P-3 respectively.

To obtain a negative control, the corresponding fragments of 3’UTR of TUSC2 and TUSC2P 3’UTR of which the miRNAs binding sites were mutated was synthesized and cloned to psiCHECK™-2 luciferase vector to obtain corresponding mutant luciferase constructs.

### Transfection

EC109 cells or TE-1 cells were seeded at six-well plates for 3’-UTR over-expression construct transduction by using Lipofectamine 2000 (Invitrogen). Transfected ESCC cells were selected by G418 to gain ESCC cells with stable over-expression of TUSC2 and TUSC2P.

EC109 or TE-1 cells were seeded in 12-well dishes and cultured for 24 h, followed by transfection of TUSC2 siRNAs or miRNAs mimics/ inhibitors using Lipofectamine 2000 (Invitrogen). The concentration for siRNAs is 200 nmol/L, and concentrations for miRNAs mimics/ inhibitors is 400 nmol/L.

### Proliferation assay

EC109 cells or TE-1 cells were transfected for 6 h, then were trypsinized, resuspended in 50 ml DMEM medium and then seeded in 5 sets of 12-well plate. Within 12-well plate, each treatment group were seeded triplicately. The cells were observed for 5 days. For each of the following day (d1, d2, d3, d4, d5), one set of plate was fixed using 10% formalin solution and kept in PBS at 4 °C. At the end of the observation, all the wells were stained with crystal violet, and then all the well were lysised with 10% acetic acid, optical density of each well was detected at 590 nm. The experiment were repeated for three times.

### Cell invasion assay

Matrigel was diluted with DMEM medium in 1:10 and coated in cell transwell membrane inserts, 100 ml matrigel for each insert. Cell transwell membrane inserts were set in 24-well plates.

EC109 cells or TE-1 cells were suspended in serum free DMEM media and seeded in transwell membrane inserts, 2× 10^3^ cell in 100 μl free DMEM media per inserts. 500 μl DMEM media with 10% FBS were added into the wells of the plate which at bottom of the inserts. Cells were incubated at 37 °C and allow cell migrate through the matrigel and the membrane in the inserts into bottom side of the membrane. After 24 h, the inserts were taken out, the cell contain DMEM media and matrigel were removed, while the cells on the bottom side of the membrane were fixed with 10% formalin solution and stained with crystal violet. Cells were photographed and counted.

### Apoptosis analysis

Sub-confluent cells were treated with 5 μM H_2_O_2_ for 40 min to induce oxidative stress. Thereafter, cells were subjected to Apoptosis assays. An Annexin V-FITC apoptosis detection kit (Biovision Inc) was used to detect apoptotic activity. Cells (1 × 10^6^) were collected and resuspended in binding buffer, and incubated with Annexin V-FITC and propidium iodide in the dark for 15 min. Annexin V-FITC binding was determined by flow cytometry (Ex = 488 nm; Em = 530 nm) using FITC signal detector (FL1) and propidium staining by the phycoerythrin emission signal detector (FL2).

### Luciferase activity assays

Luciferase activity assays were performed using a dual-luciferase reporter system developed by Promega. In brief, EC109 cell were cultured in 24-well plate at a density of 3 × 10 ^4^ cells per well. Cells were cultured at 37 °C for 24 h, TUSC2 and TUSC2P luciferase constructs or corresponding mutant constructs were co-transfected with miRNA mimics, or miRNAs controls, respectively using Lipofectamine 2000. After 12 h, cells were collected and lysed. Luciferase activity was measured using the Dual-Luciferase reporter assay system (Promega) according to the manufacturer’s instructions.

### RNA analysis

Quantification of TUSC2 mRNA transcripts was performed by SYBR Green quantitative real-time PCR using the ABI Prism 7500 sequence detection system (Applied Biosystems) with normalization to the expression of GAPDH. MiRNAs were detected by specific stem-loop primers using miScript Reverse Transcription Kit (Qiagen) and miScriptSYBR Green PCR Kit (Qiagen) with normalization to the expression of human-U6RNA. All primer sequences are provided (see Additional file 1).

### Western blot

EC109 cells or TE-1 cells were collected and lysed. Protein concentration was determined by BCA assay kit. Total protein (20 mg/well) was subjected to 12% SDS–PAGE and transferred to a nitrocellulose membrane, and hybridized with anti-TUSC2 (Abcam, Ab70182) at a dilution of 1:500 at 4 °C overnight. Secondary antibodies (Abcam, ab98488) were dialuted at 1:2,000 and incubated with the membranes at room temperature for 2 h. After secondary antibody incubation, the blot was washed and detected using ECL kit (Millipore) in autoradiography.

β-actin (Abcam, ab179467) was used to confirm equal sample loading.

### Statistical analysis

Mann–Whitney test or Student *t* test were used for were used to compare quantitative data among groups using SPSS (version 16.0; SPSS Inc., Chicago, IL). TUSC2 or TUSC2P expression intensity in tissue sample were transformed into log10, and correlations between TUSC2 or TUSC2P expression in tissue were calculated using Spearman rank correlation coefficients. For survival analysis, TUSC2 and TUSC2P expression levels in human esophagus cancer were classified into low and high subgroups according to their median expression value. The survival rate was calculated using the Kaplan Meier method, and the log rank test was performed for significance test of TUSC2 and TUSC2P subgroups. All tests were considered significant at *P* value < 0.05.

## Results

### TUSC2 and TUSC2P shared the same targeting miRNAs

**Fig. 1:**
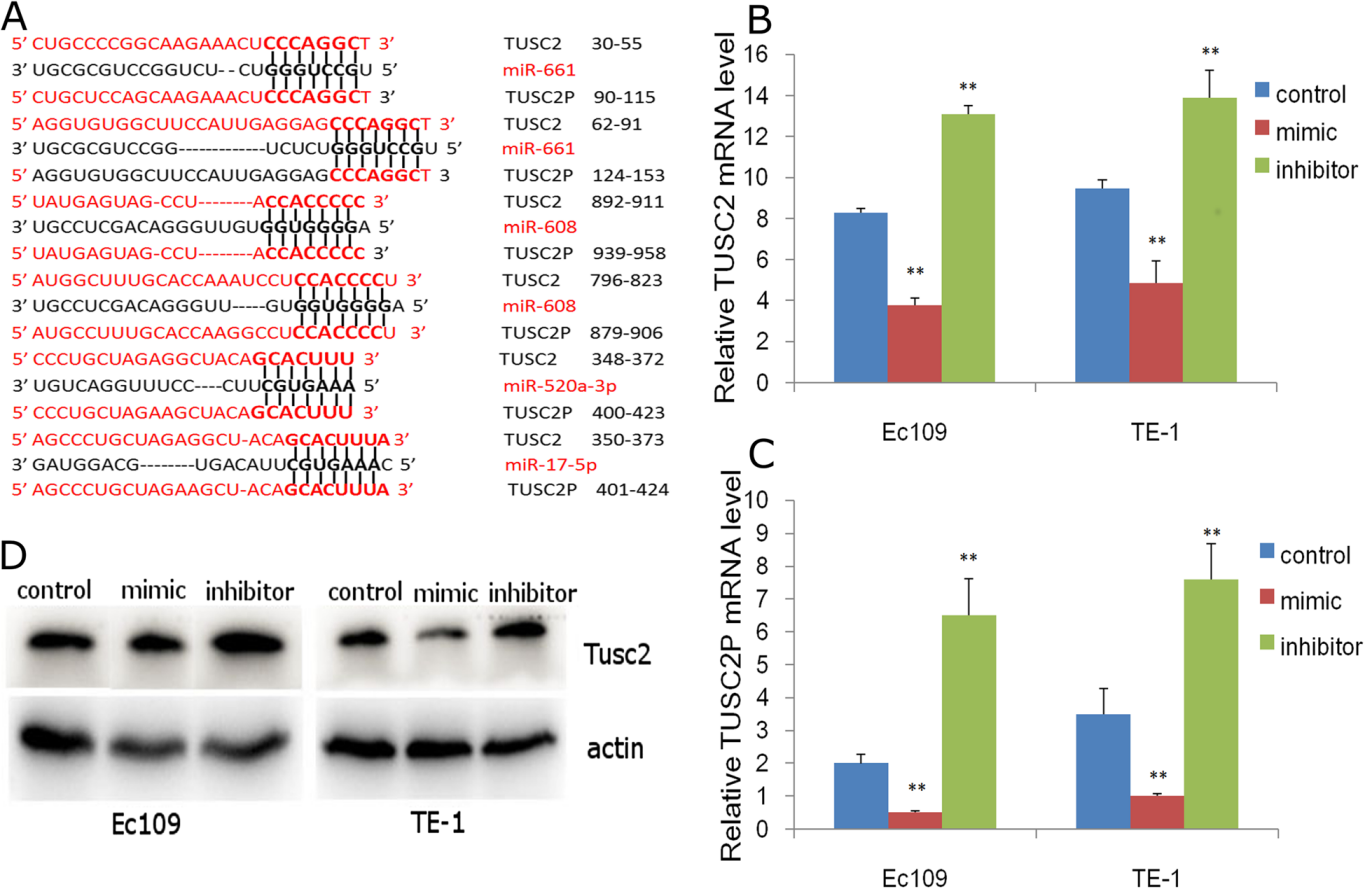
TUSC2P is targeted by TUSC2-targeting miRNAs. **a** Binding of TUSC2 targeting miRNAs to TUSC2P. Seeds and seed matches, bold; canonical pairings, solid lines; **b**, **c** TUSC2-targeting miRNA mimics decrease while miRNA inhibitors increase TUSC2 and TUSC2P mRNA abundance in ESCC cell line Ec109 and TE-1. **d** TUSC2-targeting miRNA mimics decrease while miRNA inhibitors increase TUSC2 protein abundance. *n* = 3, **P < 0.01

The DNA sequence of pseudogene TUSC2P is highly homologous to its corresponding gene TUSC2, with only eighteen mismatches for the coding sequence. TUSC2P possesses a 3’UTR that is about 1.2 kilobase, which shared 89% homology with the 3’UTR of TUSC2 (see Additional file 2). When analyzing the sequence of the TUSC2P and TUSC2 3’UTR, miRNA-17-5p, miRNA-608, miRNA-661, miRNA-520a-3p were found to poss conserved binding sites for TUSC2P and TUSC2 (Fig. 1a). Among these potential targeting miRNAs, some of them exhibit more than one potential binding site.

**Fig. 2:**
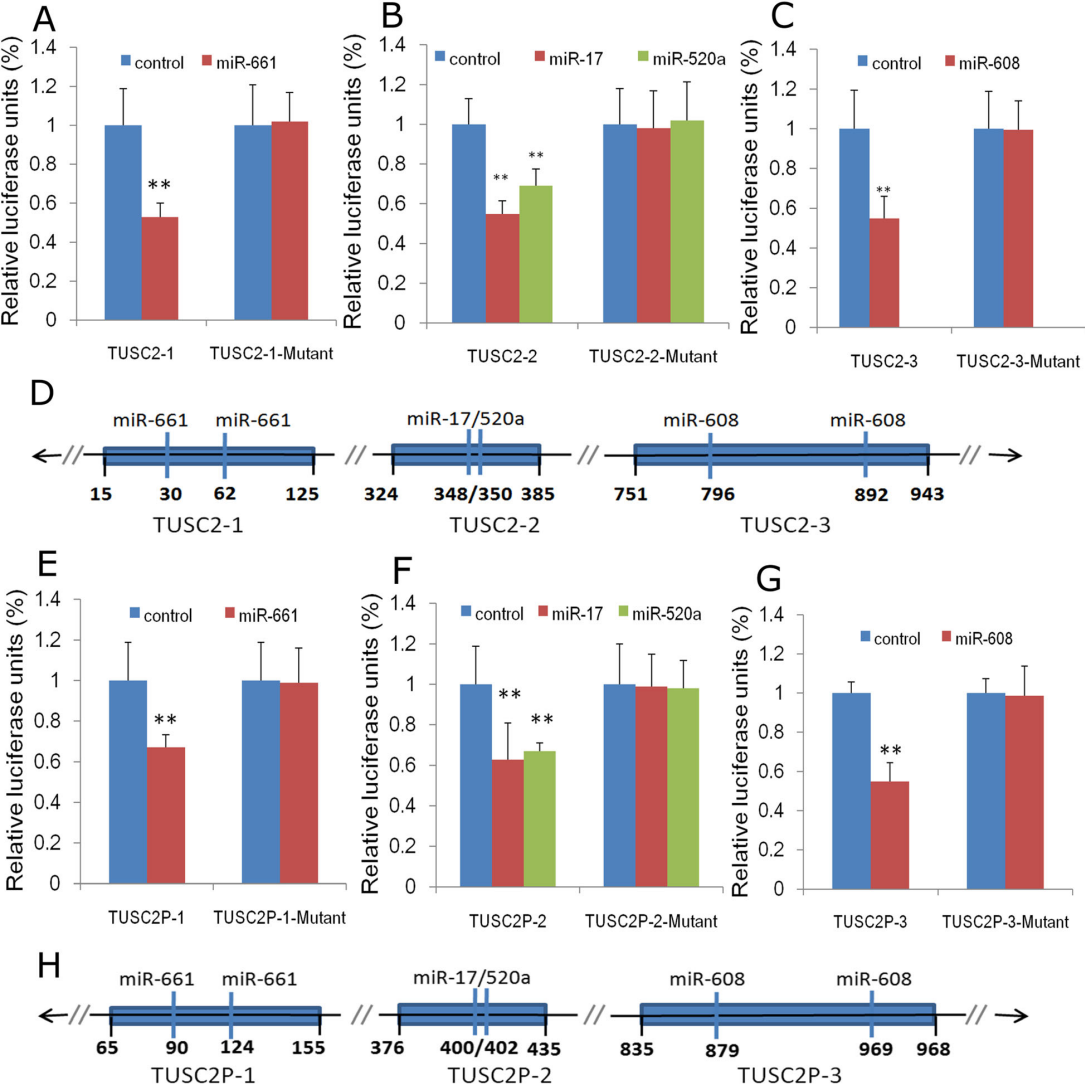
Confirmation of TUSC2 and TUSC2P targeting by luciferase activity assays. **a**-**d** Fragments of TUSC2 harbouring different target sites of miR-661 (**a**), miR-17-5p/520a-3p (**b**), miR-608 (**c**) were cloned into the luciferase reporter vector psiCHECKTM-2(D). Ec109 cells were co-transfected with one of these miRNAs and the luciferase constructs or a mutant constructs, in which the miRNA target sites were mutated. Luciferase activity assays indicated that all four miRNAs repressed luciferase activities when it harboured the corresponding TUSC2 fragment, which was reversed when the potential miRNA target site was mutated; **e**-**h** Luciferase activity assays indicated that all four miRNAs repressed luciferase activities when it harboured the corresponding TUSC2P fragment, which was reversed when the potential miRNA target site was mutated; *n* = 3, **P < 0.01

To explore the potential targeting regulation of these miRNAs on both TUSC2 and TUSC2P in ESCC. In EC109 and TE-1 esophageal cancer cells, transient transfection of a pool of miRNA mimics suppressed both TUSC2 and TUSC2P mRNA abundance (Fig. 1b and c), as well as TUSC2 protein abundance (Fig. 1d). In these ESCC cells, a pool of miRNA inhibitors de-repressed both TUSC2 and TUSC2P transcript levels (Fig. 1b and c), as well as TUSC2 protein expression (Fig. 1d). Luciferase activity assays indicated that the miRNA–TUSC2 (Fig. 2a–d) and miRNA–TUSC2P (Fig. 2e–h) interaction was direct, which indicate that TUSC2 and TUSC2P are targeted by common miRNAs and in the post-transcriptional regulation manner.

### Expression of TUSC2P suppresses tumour activity in ESCC

The TUSC2P 3’-UTR over-expression construct was generated to express TUSC2P 3’UTR, and was then transfected into EC109 cells or TE-1 cells to examined the ability of TUSC2P 3’ UTR as decoy of TUSC2 -targeting miRNAs.

**Fig. 3:**
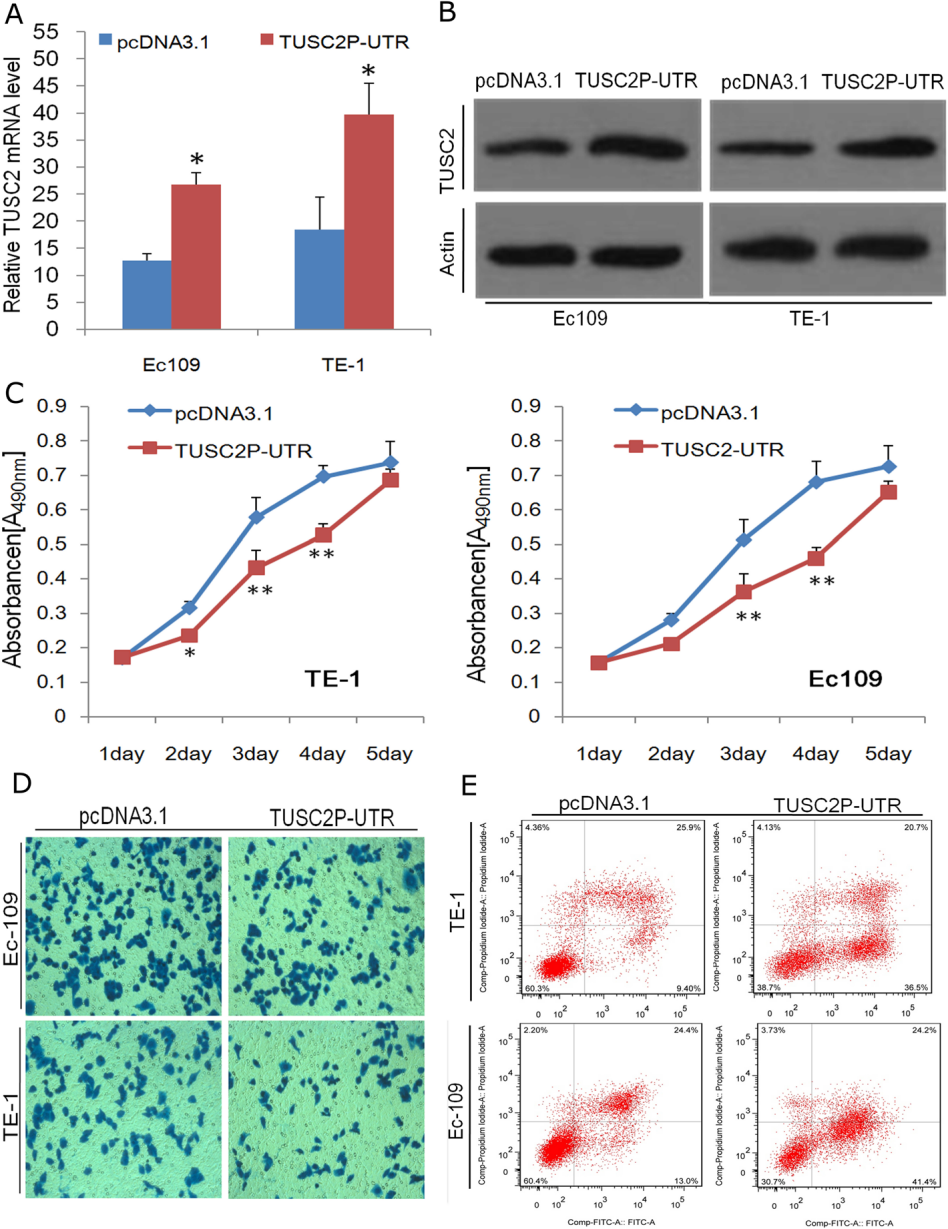
TUSC2P 3’ UTR exerts a tumour suppressive function. **a**, **b** TUSC2P 3’UTR transfected Ec109 and TE-1 cell showed increased TUSC2 mRNA and protein levels. **c**-**e** TUSC2P 3’UTR-transfection decreased proliferation rate and invasion, while promoted apoptosis in Ec109 and TE-1 cell. *n* = 3, *P < 0.05,**P < 0.01

Indeed, TUSC2P 3’UTR over expression resulted in a de-repression of both TUSC2 transcript and protein in EC109 and TE-1 esophageal cancer cells (Fig. 3a and b). Consistent with elevated TUSC2, TUSC2P 3’UTR over expression can induce growth (Fig. 3c) and invasion inhibition (Fig. 3d), and promote apoptosis (Fig. 3e).

**Fig. 4:**
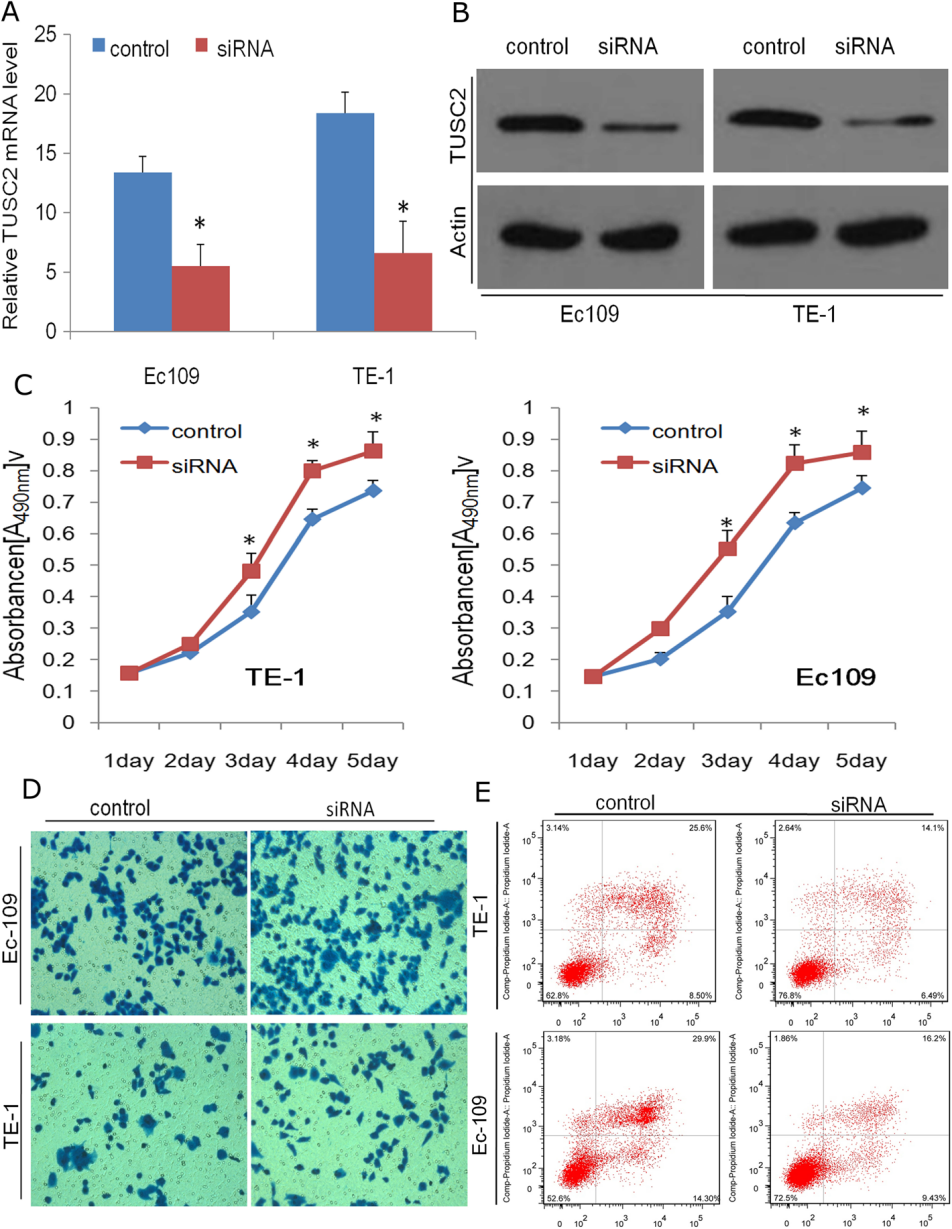
Silencing of TUSC2 and TUSC2P exerts a tumour promotion function. siRNA pools for TUSC2 which can bind to common sequences in both TUSC2 and TUSC2P were transfected in Ec109 and TE-1 cell. **a**, **b** siRNAs transfection decreased TUSC2 mRNA and protein levels. **c**-**e** siRNAs transfection increased proliferation rate and invasion, while reduced apoptosis in Ec109 and TE-1 cell. *P < 0.05

To examine the consequences of TUSC2 and TUSC2P down-regulation, siRNAs for TUSC2 (si-TUSC2/ TUSC2P) which can suppress both TUSC2 and TUSC2P expression was transfected into EC109 cells or TE-1 cells Transfection of si-TUSC2/ TUSC2P in EC109 and TE-1 cell reduced TUSC2 expression (Fig. 4a and b), and thus accelerated cell proliferation (Fig. 4c) and invasion (Fig. 4d), decelerated apoptosis (Fig. 4e), indicating that TUSC2 and its pseudogene have important roles in tumor cell biology.

### The tumour suppressive function of TUSC2P is dependent on miRNA binding

**Fig. 5:**
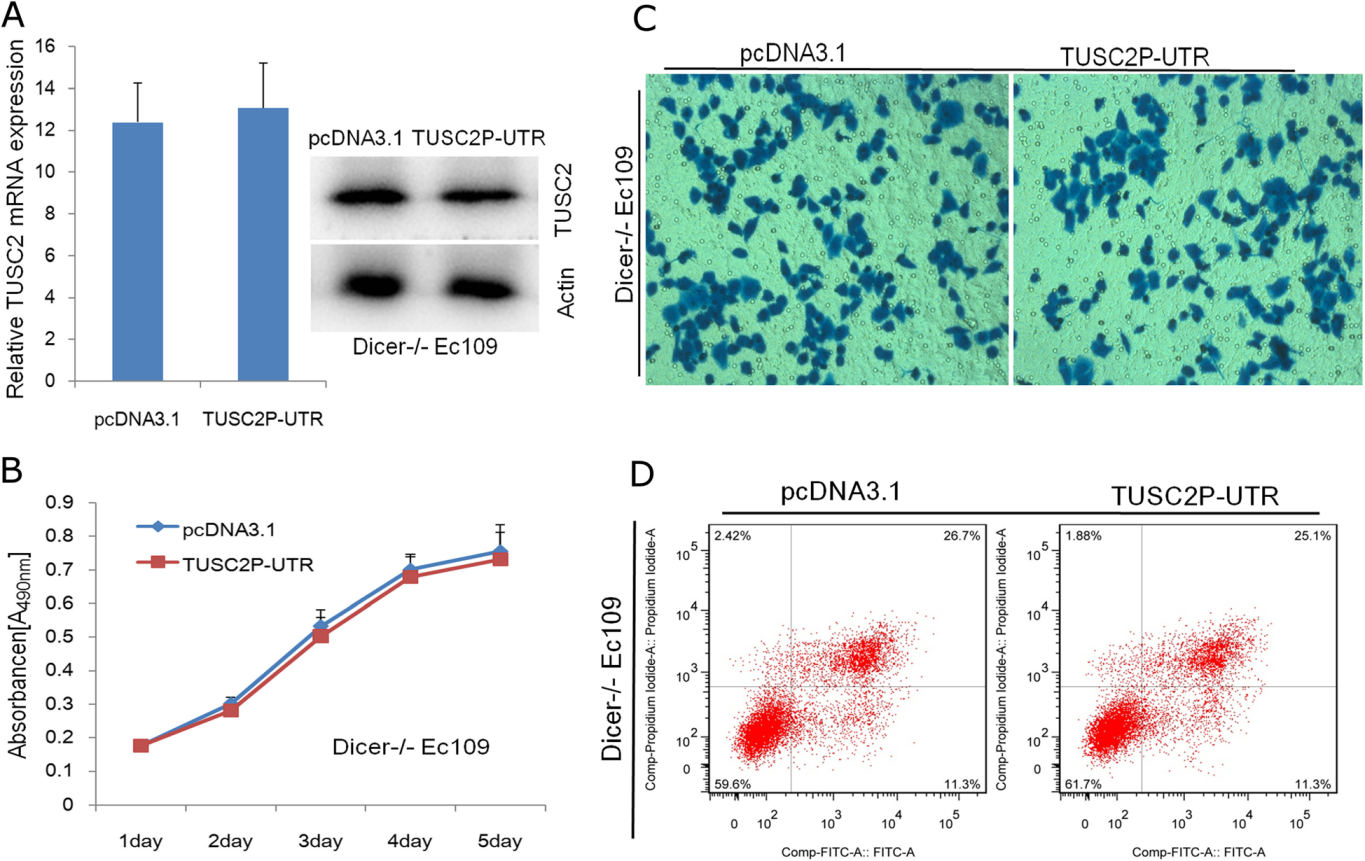
The tumour suppressive function of TUSC2P is dependent on miRNA binding. TUSC2P 3’ UTR or empty vector was transfected in parental Ec109 or Ec109 DICER−/− cells. **a** De-repression of TUSC2 abundance by TUSC2P 3’ UTR over-expression was blunted in DICER −/− EC109 cells. **b**-**d** Effects of TUSC2P 3’UTR over expression on growth (**b**), invasion (**c**) and apoptosis (**d**) were also blunted in DICER −/− EC109 cells (*n* = 3)

DICER ^−/−^ EC109 cells were established to investigate the potential mechanism by which TUSC2P can regulate TUSC2 expression. DICER is a critic enzyme for miRNA maturation. In DICER ^−/−^ EC109 cells, expression of DICER was silenced, leading to decreased expression level of miRNAs compared to control EC109 cells (see Additional file 3). We observed that the de-repression of TUSC2 abundance by TUSC2P 3’ UTR over-expression was blunted under absent expression of miRNAs (Fig. 5a), which supported that regulation of TUSC2 by 3’UTR of TUSC2P requires mature miRNAs. Consistent with blunted TUSC2 de-repression, effects of TUSC2P 3’UTR over expression on growth (Fig. 5b), invasion (Fig. 5c) and apoptosis (Fig. 5d) were also blunted in DICER −/− EC109 cells.

### Expression and losses of TUSC2 and TUSC2P in ESCC patients

**Fig. 6:**
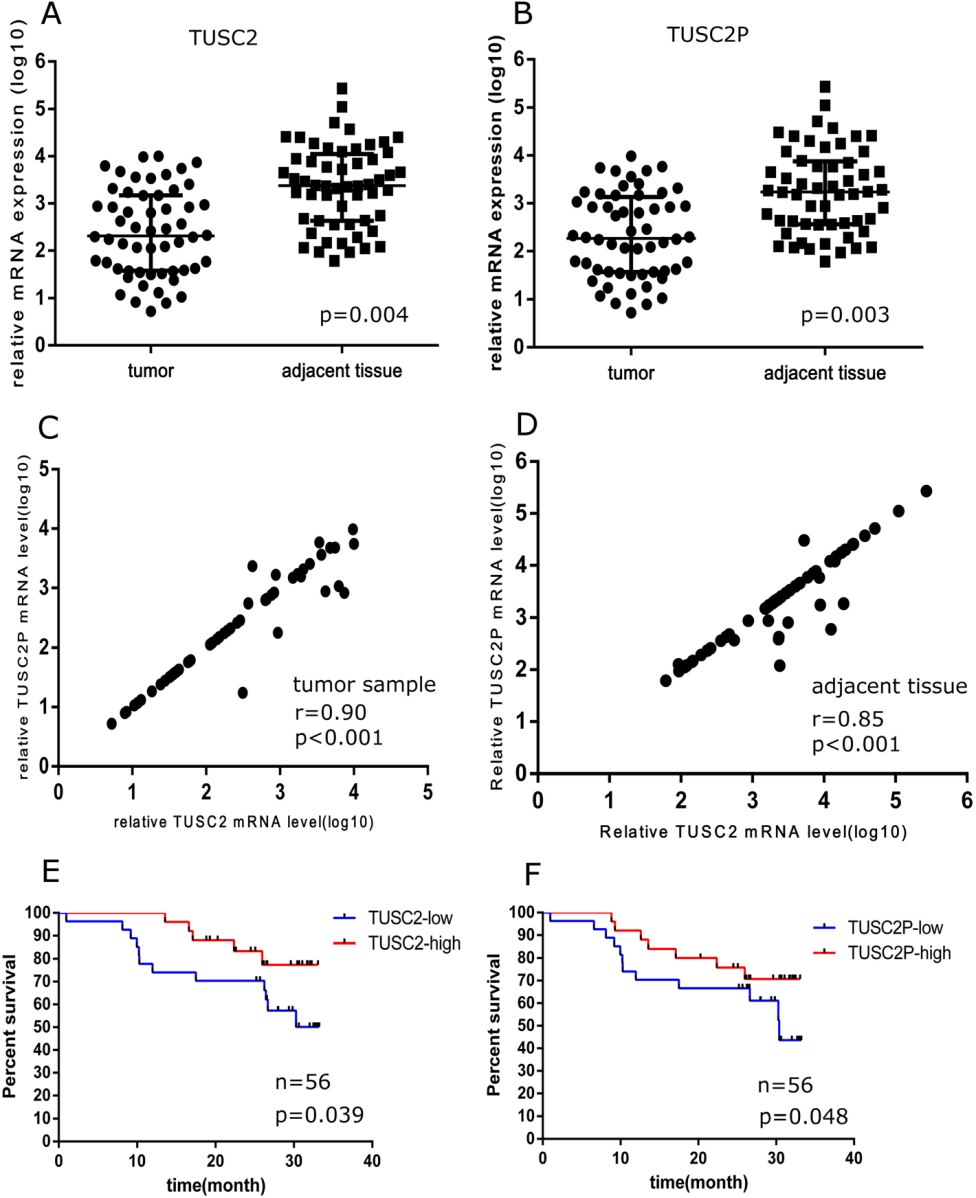
TUSC2 and TUSC2P were depressed in ESCC tissues, and decreased levels of TUSC2 and TUSC2P indicate worse ESCC prognosis. **a**, **b** The expression level of TUSC2 and TUSC2P is lower in ESCC tissues compared with adjacent normal tissues (*n* = 56). **c**, **d** TUSC2P expression is highly related to TUSC2 in both ESCC tissues and adjacent normal tissues (*n* = 56). **e**, **f** Kaplane-Meier survival curves according to the relative expression level of TUSC2 and TUSC2P in 56 ESCC patients. Decreased level of TUSC2 and TUSC2P indicate worse ESCC prognosis

TUSC2 and TUSC2P expression was detected in tumor samples and matched adjacent normal tissue from ESCC patient (*n* = 56), and then validated in oral cancer samples (*n* = 49), using qRT-PCR. Both TUSC2 and TUSC2P expression levels were decreased in esophagus tumour samples compared with matched adjacent normal tissue (Fig. 6a and b). Additionally, TUSC2 and TUSC2P expression showed highly correlation (*r* = 0.90, *P* < 0.001 and *r* = 0.85, *P* < 0.001, respectively) in both adjacent tissue and esophagus tumour samples, which suggests that they may be co-regulated in vivo (Fig. 6c and d). In further, higher level of TUSC2 and TUSC2P expression predicted better survival in esophagus cancer patients (Fig. 6e and f). Similar pattern was observed in oral cancer samples (see Additional file 4). This finding supports our molecular observations that TUSC2 and TUSC2P exert important role in tumor cell biology, and TUSC2P can regulate TUSC2 expression.

## Discussion

Pseudogenes are fragments of DNA with variation in sequence relative to the parent coding gene. Pseudogenes have lost at least some functionality, relative to the complete gene, in cellular gene expression or protein-coding ability. Although not being fully functional, pseudogenes are similar to other noncoding RNA, which can perform regulatory functions. Many pseudogenes have important roles in normal physiologic and abnormal pathologic process [17, 18]. Similarly to other non-coding RNAs, pseudogenes can affect the function and/or expression of their parental genes or other coding genes through mechanisms as following:1) Producing antisense transcripts thus can completely bind to its parental gene and act as endogenous siRNAs [19, 20]; 2) Functioning a competing endogenous RNAs, which can regulate the stability and translation of other RNA transcripts by competing for shared microRNAs [21, 22]; 3) Encoding shortened proteins or peptides, which exert certain functions in cell [23, 24]. Pseudogene-mediated miRNA decoys is the most functional way since all transcribed pseudogenes can in principle compete with cognate genes for miRNA binding, only few pseudogenes undergo antisense transcription or produce functional protein. However, very few pseudogenes have been functionally well elucidated so far.

TUSC2P was a newly discovered and poorly elucidated pseudogene [25]. The only related study reported that TUSC2P may exert tumor suppression role in prostate tumor [26]. However, the involvement of TUSC2P in the development of ESCC have yet not been well characterized. In the present study, for the first time, we reported a functional role for TUSC2P is relevant to TUSC2 biology in esophageal carcinoma as minute changes in TUSC2 can have tumorigenic consequences. In our analysis, we found that TUSC2P derepress TUSC2 expression by competing with miRNA binding. Loss of TUSC2P and TUSC2 can lead to accelerated proliferation and invasion, decelerated apoptosis in esophageal cancer cell, suggesting TUSC2P is a bona fide tumor suppressor gene. Actually, TUSC2P is not the first functional pseudogene as miRNA decoy, which can regulated corresponding cognate genes. Pseudogene PTENP1 was mostly elucidated and was reported to regulate cellular levels of PTEN and exert a growth-suppressive role in prostate cancer cell [27]. Pseudogene PHBP1 promotes esophageal squamous cell carcinoma proliferation by increasing its cognate gene PHB expression [28]. Pseudogene GBAP1 regulates the glucocerebrosidase gene GBA by competively binding miR-22–3p [29].

Additionally, TUSC2P is repressed in ESCC tissues compared with adjacent normal tissues, and that expression level of TUSC2P and corresponding cognate gene TUSC2 is associated with survival outcome of ESCC.

Accumulating evidence showed that pseudogenes usually expressed in a cancer specific pattern [17, 27, 30]. They have shown differential expression profile between tumor species and normal control tissues, or sometimes their expression can be tumor tissue specific [31, 32]. To date, pseudogene detection has been neglected in most of the studies about their parental genes. However, more and more pseudogenes have been identified, and they exhibit cancer specific expression pattern and are proven to be related to cancer. Thus makes it as a potential diagnostic and prognostic indicator [33–36]. For example increased levels of OCT4 pseudogene POU5F1B has been reported to promote tumor growth and predict poor prognosis in stage IV gastric cancer patients [37]. Another OCT4 pseudogene OCT4-pg4 can competitively bind to miRNA-145 thus regulate OCT4 expression. Additionally OCT4-pg4 is over expressed in hepatocellular carcinoma and predict poor prognosis [38]. In light of this, pseudogenes can not only play structural and functional roles in the tumorigenesis, but also in disease diagnosis and prognosis. Therefore, a better understanding of pseudogenes expression change may provide important clues for both etiology and prognosis of cancer.

## Conclusions

In summary, we provided a better understanding of the biology of ESCC carcinogenesis by TUSC2P. We investigate that the TUSC2P was significantly down regulated in ESCC tissues and increased expression of TUSC2P might play a tumor repressive role in ESCC carcinogenesis by acting like ‘endogenous competitors’ of miRNAs, consequently de-repressed TUSC2 mRNA. All of these findings illustrate the important roles of TUSC2P in ESCC carcinogenesis and the potential role of TUSC2P as a novel biomarker for ESCC.

## Additional files

Additional file 1: Primers of mRNA and miRNA. Primers sequences of mRNA and miRNA used for PCR in the manuscript. (DOCX 16 kb)

Additional file 2: Sequence Alignment of TUSC2 and TUCS2P. TUSC2P possesses a 3’UTR that is about 1.2 kilobase, which shared 89% homology with the 3’UTR of TUSC2. (DOCX 17 kb)

Additional file 3: Silenced expression of miRNAs in DICER −/− EC109 cells. Expression of miR-17-5p, miR-520a-3p, miR-608, miR-661 in were silenced DICER −/− EC109 cells. (JPG 53 kb)

Additional file 4: TUSC2 and TUSC2P were more lowly expressed in oral cancer tissues, and decreased levels of TUSC2 and TUSC2P indicate worse oral cancer prognosis. (A-B) The expression level of TUSC2 and TUSC2P is lower in oral cancer tissues compared with adjacent normal tissues (*n* = 49). (C-D) TUSC2P expression is related to TUSC2 in both oral cancer tissues and adjacent normal tissues (n = 49). (E-F) Kaplane-Meier survival curves according to the relative expression level of TUSC2 and TUSC2P in 49 oral cancer patients. Decreased level of TUSC2 and TUSC2P indicate worse oral cancer prognosis. (JPG 797 kb)

## Abbreviations

ESCC: Esophageal squamous cell carcinoma
GBA: Glucocerebrosidase
OCT4: Octamer-binding protein 4
PHB: Prohibitin
POU5F1B: POU domain class 5 transcription factor 1B
PTEN: Phosphatase and tensin homolog
TUSC2: Tumor suppressor candidate-2
TUSC2P: Tumor suppressor candidate-2 pseudogene
