# Supplementary material for: TUSC2P suppresses the tumor function of esophageal squamous cell carcinoma by regulating TUSC2 expression and correlates with disease prognosis
Source: BMC Cancer. 2018 Sep 15;18:894. doi: 10.1186/s12885-018-4804-9 (PMC6139140; doi:10.1186/s12885-018-4804-9)
Supplement: Supplementary file 1 — Primers of mRNA and miRNA. Primers sequences of mRNA and miRNA used for PCR in the manuscript. (DOCX 16 kb) [file 12885_2018_4804_MOESM1_ESM.docx]

Table S1 Primers of mRNA and miRNA

| **Name of mRNA** | **Sequence of primer (5’ to 3’)** |
| --- | --- |
| Human-GAPDH-F | AAGGCTGGGGCTCATTTGCAG |
| Human-GAPDH-R | GATGTTCTGGAGAGCCCCGCG |
| Human-TUSC2-F | GGGCTCGAGCCCTGGGAGGTGGCAGACAGAAGC |
| Human-TUSC2-R | GGGCCCACGCGTGTAACGGGCATTCCTGACTG |
| Human-TUSC2P-F | CCGAGCTCCATCCATGTGGATTTCCCTCTG |
| Human-TUSC2P-R | CCACGCGTTTCAGAGTTGTGGCCCCAGCCTG |
| Human-U6-F | GTGCTCGCTTCGGCAGCACATATAC |
| Human-U6-R | AAAAATATGGAACGCTTCACGAATTTG |
| **Name of miRNA** | **Sequence of primer(5’ to 3’)** |
| Hsa-miRNA-608 | AGGGGTGGTGTTGGGACAGCTCCGT |
| Hsa-miRNA-661 | TCTCTGGCCTGCGCGTAAA |
| Hsa-miRNA-17-5p | CAAAGTGCTTACAGTGCAG |
| Has- miRNA-520a-3p | AAAGTGCTTCCCTTTGGACTGT |
