# Supplementary material for: TUSC2P suppresses the tumor function of esophageal squamous cell carcinoma by regulating TUSC2 expression and correlates with disease prognosis
Source: BMC Cancer. 2018 Sep 15;18:894. doi: 10.1186/s12885-018-4804-9 (PMC6139140; doi:10.1186/s12885-018-4804-9)
Supplement: Supplementary file 2 — Sequence Alignment of TUSC2 and TUCS2P. TUSC2P possesses a 3’UTR that is about 1.2 kilobase, which shared 89% homology with the 3’UTR of TUSC2. (DOCX 17 kb) [file 12885_2018_4804_MOESM2_ESM.docx]

Table S2 Sequence Alignment of TUSC2 and TUCS2P

Query 101 ACTCCTGCATCCATGTGGATTTCCCTCTGATCCTCTATGAGATGTGAGCCTCGGA---AG 157

|| || ||||||| |||||||||||| |||||||||||||| ||||| ||| ||| |

Sbjct 426 ACCCCCGCATCCACGTGGATTTCCCTGTGATCCTCTATGAGGTGTGACCCTGGGAGGTGG 485

Query 158 CAGACAATAGCACCTCCTGCTCCAGCAAGAAACTCCCAGGC----TCAAGGTGTGGCTTC 213

|||||| |||||| ||||| || ||||||||||||||||| |||||||||||||||

Sbjct 486 CAGACAGAAGCACCCCCTGCCCCGGCAAGAAACTCCCAGGCTCAATCAAGGTGTGGCTTC 545

Query 214 CATTAAGGAGCCCAGGCTGGGGCCACAACTCTGAATAACCTCTGTTGGCACATAACCTTC 273

|||| |||||||||||||||||||||||| |||||||| |||||||||| ||||||||||

Sbjct 546 CATTGAGGAGCCCAGGCTGGGGCCACAACCCTGAATAAACTCTGTTGGCCCATAACCTTC 605

Query 274 AGCTGTGAGTGGTTCAGTCCTGTGATAttggttgggtgttggtttgtgtgtggacaagag 333

||||||||| || || |||| |||||||||||||||||||||||||||||||||||

Sbjct 606 AGCTGTGAGCGGGTCGGTCCCACAGTATTGGTTGGGTGTTGGTTTGTGTGTGGACAAGAG 665

Query 334 gtggttggtggctggtggAGGCTAATGGCAGAGTTAGCCCCCTACTCTCCCCAGCCACCC 393

||||||||||| ||||| |||||||||||||||||||| ||| |||||||| ||||||||

Sbjct 666 GTGGTTGGTGGGTGGTGAAGGCTAATGGCAGAGTTAGCACCCCACTCTCCCAAGCCACCC 725

Query 394 CTGCAAGAAGCATGGCAGGGCATATACCAGTCAGGAATGCCCAGTACCTGGTTCCTTGCC 453

||||||| |||| |||||||||||||||||||||||||||| ||||||||||||||||

Sbjct 726 CTGCAAGCAGCACAGCAGGGCATATACCAGTCAGGAATGCCCGTTACCTGGTTCCTTGCC 785

Query 454 TGGTCTGCTTTCCTCCACGTTTGCCTGGGGCCTAACCCTGCTAGAAGCTACAGCACTTTA 513

|||||||||||| |||| |||||||||||||||| |||||||||| ||||||||||||||

Sbjct 786 TGGTCTGCTTTCTTCCAAGTTTGCCTGGGGCCTAGCCCTGCTAGAGGCTACAGCACTTTA 845

Query 514 TAAGCAAGGTTTGCCTTCTTCCAGCTCCTAGGCTGTGGGTGCTGTATACAATTAGGAACT 573

||||||||| ||| |||||||||| ||||||||||||| |||||||||| ||||||||

Sbjct 846 CAAGCAAGGTATGCTTTCTTCCAGCCCCTAGGCTGTGGGCACTGTATACAAGTAGGAACT 905

Query 574 TCCTTTCCTTCACTTCCCTTTTAACCCCTAGTCAGAGCATTTCAGCTGTTGTTTGCTACT 633

|||||||||||||||||||||||||||||||||||||||||||||| ||| | |||||

Sbjct 906 TCCTTTCCTTCACTTCCCTTTTAACCCCTAGTCAGAGCATTTCAGCCGTT-T--GCTACC 962

Query 634 TA-ATTCCTCCCATGTTGGACAGAGGCTGGGGGCAGTGCCAGCCTGATTCTTCCCACCTA 692

| |||||||| ||||||||||||||||||||||||||||||||||||||||| |||||

Sbjct 963 TCGATTCCTCCTGTGTTGGACAGAGGCTGGGGGCAGTGCCAGCCTGATTCTTCCGACCTA 1022

Query 693 CCTGCCATCTGTTCCCACCTTCAGATGGATGGACAGTTTGCTGGCTGTTGATAGGAGTGG 752

|||||||| ||||||| ||||||||||||||||||||||||||||| |||||||||||||

Sbjct 1023 CCTGCCATTTGTTCCCGCCTTCAGATGGATGGACAGTTTGCTGGCTATTGATAGGAGTGG 1082

Query 753 GGACTGGACAGGGTCTTCACCCTCTACCAAGGGCTGGGCTGATCCCCCTACTACAACTAG 812

||||||| ||| |||| ||||||||| ||||||||||||||||||||||| ||||||

Sbjct 1083 GGACTGGGTGGGGGCTTCTCCCTCTACCCAGGGCTGGGCTGATCCCCCTACTGCAACTAA 1142

Query 813 TTGTTGcccccc--c-----acccccAGTTGAGGAGTTGACAGGGTGCAGGCTGGGGTCA 865

||||||||||| | |||||||||||||||||||| || ||||||||||||||||

Sbjct 1143 CTGTTGCCCCCCAACCCCGAACCCCCAGTTGAGGAGTTGAGAGAGTGCAGGCTGGGGTCA 1202

Query 866 GGACAGGCTGTGGATGCTTGTGTCTATAGGGAGTTACTCCAACCCACCTATTCTGTCTAA 925

|||||||||| ||||||||||| |||| ||||||||||||||||||||||||||||||||

Sbjct 1203 GGACAGGCTGCGGATGCTTGTGCCTATGGGGAGTTACTCCAACCCACCTATTCTGTCTAA 1262

Query 926 TCCCCCATGCCTTTGCACCAAGGCCTCTACCCTTCCAATTGGGAGGGAACTATTCACCAC 985

|| ||||| ||||||||||| |||| |||| |||||||||||||| ||| ||||||||

Sbjct 1263 TCT-CCATGGCTTTGCACCAAATCCTCCACCCCTCCAATTGGGAGGGGACTGTTCACCAC 1321

Query 986 CCTGTGGTAAGGGACAACATCCTAAGGCTGGTGCCAATAGTTATGAGTAGCCTACCACCC 1045

| ||||||||||||||||| |||||||||||||||| |||||||||||||||||||||||

Sbjct 1322 CTTGTGGTAAGGGACAACACCCTAAGGCTGGTGCCAGTAGTTATGAGTAGCCTACCACCC 1381

Query 1046 CCTTCCCCTACAGTAACCTCCACCCCTTCAGGATGAGTCAAGGGAAAGTACTGGAGCCGC 1105

|| |||| |||||||||| ||||||||||||||| ||||||||||||| ||| || || |

Sbjct 1382 CC-TCCCTTACAGTAACCCCCACCCCTTCAGGATCAGTCAAGGGAAAGCACTAGAACCCC 1440

Query 1106 TGGGTATGAAAAGAAAGGAGGGAAAAATCATAAAAGGAATA---------TGAAGGTTTG 1156

|||||| | |||||||||||||||||| ||||||||||||| ||||||||||

Sbjct 1441 TGGGTAGGGAAAGAAAGGAGGGAAAAACCATAAAAGGAATACTTATAATGTGAAGGTTTG 1500

Query 1157 TAAATAGTCCACGATGATGTCATGGCAGAGTCTGATTTCTATATAGAGGTAACTTaaaaa 1216

||||||||||| ||||||||| |||||||||||||||||||||||||||| ||||

Sbjct 1501 TAAATAGTCCATGATGATGTCGTGGCAGAGTCTGATTTCTATATAGAGGTGACTTTTTTT 1560

Query 1217 aaaa-TACTGTGCAAGATCTGTTCTTCTAGAGTGTGGGAAATGGCTTGCGGAGGGTGGCC 1275

|| ||||||||||| ||||| |||||| | |||||||||||||||| ||||| |||||

Sbjct 1561 TTAAGTACTGTGCAAGCTCTGTGCTTCTATAATGTGGGAAATGGCTTGGGGAGGATGGCC 1620

Query 1276 CCCAGCCTAGAAAGACTATTGTGCTATTTGTTCAATTTCAATAAAATGATTTATAGATCC 1335

|| ||| ||| |||||| ||||| |||||||||||||||||||||||||||| |||||||

Sbjct 1621 CCTAGCTTAGGAAGACTGTTGTGTTATTTGTTCAATTTCAATAAAATGATTTGTAGATCC 1680

Query 1336 TG-AAAATAAATAAA 1349

|| |||| ||| |||

Sbjct 1681 TGCAAAAAAAAAAAA 1695

Note: Upper lane:TUCS2P mRNA; Lower lane:TUSC2 mRNA.
