# Supplementary figures and images for: TUSC2P suppresses the tumor function of esophageal squamous cell carcinoma by regulating TUSC2 expression and correlates with disease prognosis
Source: BMC Cancer. 2018 Sep 15;18:894. doi: 10.1186/s12885-018-4804-9 (PMC6139140; doi:10.1186/s12885-018-4804-9)

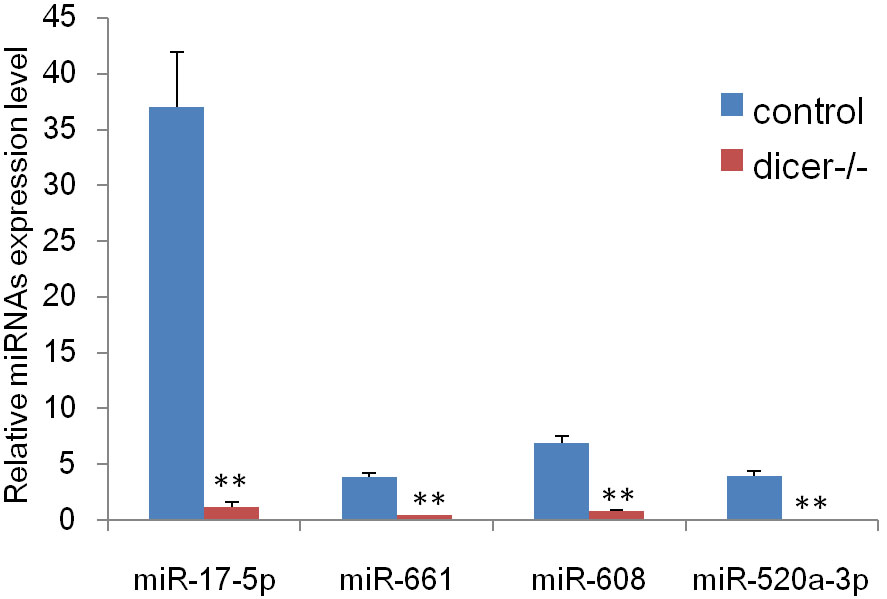

Supplement: Supplementary file 3 — Silenced expression of miRNAs in DICER −/− EC109 cells. Expression of miR-17-5p, miR-520a-3p, miR-608, miR-661 in were silenced DICER −/− EC109 cells. (JPG 53 kb) [file 12885_2018_4804_MOESM3_ESM.jpg]

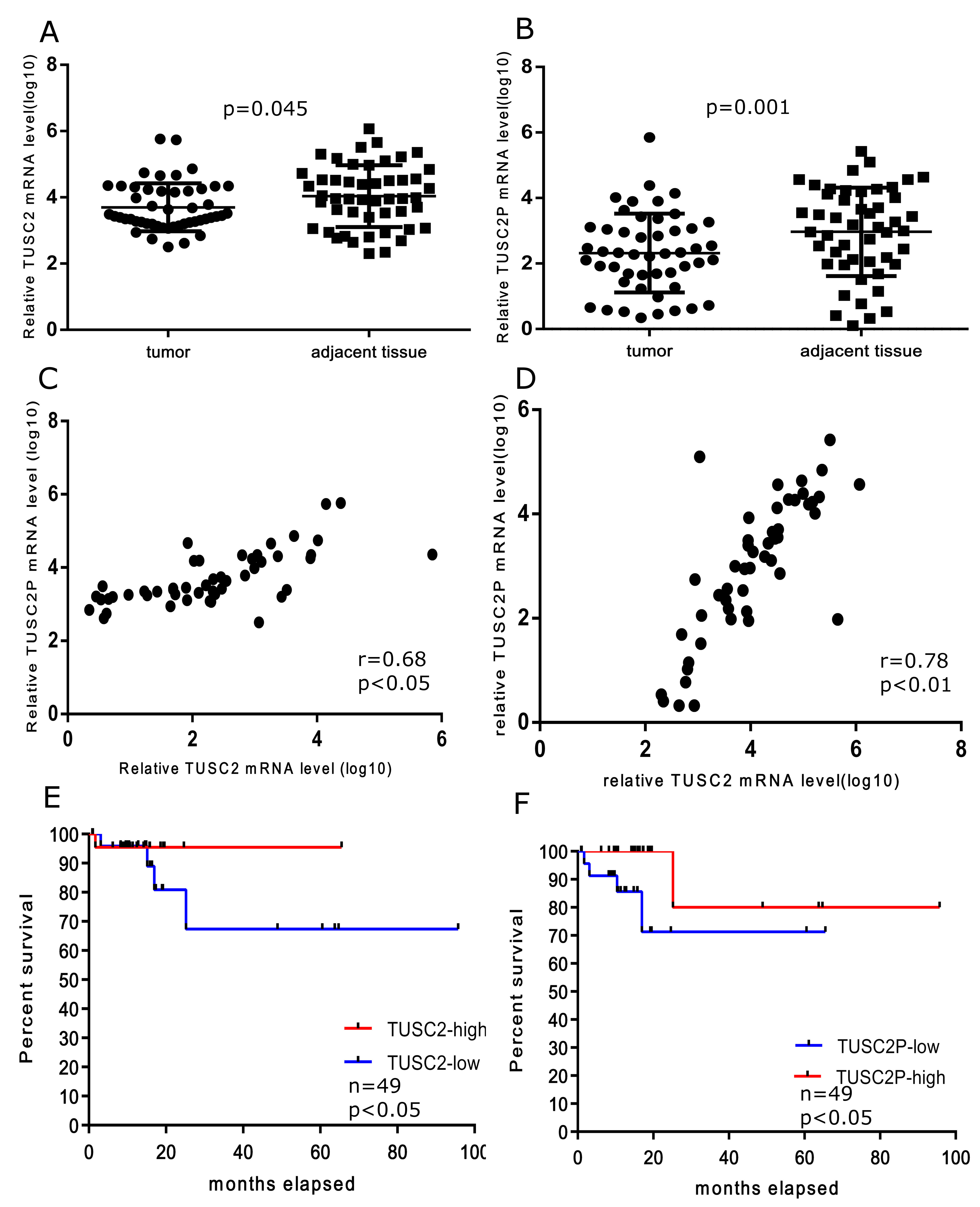

Supplement: Supplementary file 4 — TUSC2 and TUSC2P were more lowly expressed in oral cancer tissues, and decreased levels of TUSC2 and TUSC2P indicate worse oral cancer prognosis. (A-B) The expression level of TUSC2 and TUSC2P is lower in oral cancer tissues compared with adjacent normal tissues (n = 49). (C-D) TUSC2P expression is related to TUSC2 in both oral cancer tissues and adjacent normal tissues (n = 49). (E-F) Kaplane-Meier survival curves according to the relative expression level of TUSC2 and TUSC2P in 49 oral cancer patients. Decreased level of TUSC2 and TUSC2P indicate worse oral cancer prognosis. (JPG 797 kb) [file 12885_2018_4804_MOESM4_ESM.jpg]
